# Metabolomic Changes in Naturally MAP-Infected Holstein–Friesian Heifers Indicate Immunologically Related Biochemical Reprogramming

**DOI:** 10.3390/metabo11110727

**Published:** 2021-10-23

**Authors:** Emma N. Taylor, Manfred Beckmann, Bernardo Villarreal-Ramos, Hans-Martin Vordermeier, Glyn Hewinson, David Rooke, Luis A. J. Mur, Ad P. Koets

**Affiliations:** 1 Institute of Biological, Environmental and Rural Sciences (IBERS), Aberystwyth University, Ceredigion SY23 3DA, UK; emt26@aber.ac.uk (E.N.T.); meb@aber.ac.uk (M.B.); bev10@aber.ac.uk (B.V.-R.); hav4@aber.ac.uk (H.-M.V.); glh14@aber.ac.uk (G.H.); 2 Centre of Excellence for Bovine Tuberculosis, Aberystwyth University, Ceredigion SY23 3DA, UK; 3 Animal and Plant Health Agency, Weybridge, Surrey KT15 3NB, UK; 4 ProTEM Services Ltd., Horsham, West Sussex RH12 4BD, UK; david.rooke@dynamicextractions.com; 5 Wageningen Bioveterinary Research, 8221 RA Lelystad, The Netherlands; 6 Population Health Systems, Faculty of Veterinary Medicine, Utrecht University, 3584 CS Utrecht, The Netherlands

**Keywords:** *Mycobacterium avium* subsp. *paratuberculosis*, metabolomics, antibody, MAP antigens, eicosanoids, inflammation

## Abstract

Johne’s disease, caused by *Mycobacterium avium* subsp. *paratuberculosis* (MAP), causes weight loss, diarrhoea, and reduced milk yields in clinically infected cattle. Asymptomatic, subclinically infected cattle shed MAP bacteria but are frequently not detected by diagnostic tests. Herein, we compare the metabolite profiles of sera from subclinically infected Holstein–Friesian heifers and antibody binding to selected MAP antigens. The study used biobanked serum samples from 10 naturally MAP-infected and 10 control heifers, sampled monthly from ~1 to 19 months of age. Sera were assessed using flow infusion electrospray–high-resolution mass spectrometry (FIE–HRMS) on a Q Exactive hybrid quadrupole–Orbitrap mass spectrometer for high-throughput, sensitive, non-targeted metabolite fingerprinting. Partial least-squares discriminant analyses (PLS-DA) and hierarchical cluster analysis (HCA) of the data discriminated between naturally MAP-infected and control heifers. In total, 33 metabolites that differentially accumulated in naturally MAP-infected heifers compared to controls were identified. Five were significantly elevated within MAP-infected heifers throughout the study, i.e., leukotriene B4, bicyclo prostaglandin E2 (bicyclo PGE2), itaconic acid, 2-hydroxyglutaric acid and N6-acetyl-L-lysine. These findings highlight the potential of metabolomics in the identification of novel MAP diagnostic markers and particular biochemical pathways, which may provide insights into the bovine immune response to MAP.

## 1. Introduction

Paratuberculosis, commonly known as Johne’s disease, is a chronic intestinal infection in ruminants, caused by *Mycobacterium avium* subspecies *paratuberculosis* (MAP). MAP is commonly transmitted via infected faeces or colostrum, but other transmission routes include in utero, semen and contaminated environments such as manure, soil or stream water [1]. The progression of MAP infections can be subdivided into an incubation period and subclinical and clinical stages [2]. Infected cattle begin shedding MAP during the subclinical stage, via faeces [3] and milk [4]. However, clinical signs, such as weight loss, diarrhoea and reduced milk yields, are absent until 2 to 6 years post-infection [5]. A UK-based examination of the financial impact of MAP infections reported a total loss of £112.89 per infected cow, including £60.57 through milk yield losses and £51.19 via voluntary culling [6]. A UK-based survey using ELISA tests estimated a herd prevalence of 34.7% (credible interval 27.6–42.5%) [7] but this is most likely an under-estimate. 

Detecting MAP infections is challenging, as only 10–15% of MAP-infected cattle display clinical signs [8], and the performance of diagnostic tests is dependent on the stage of infection [9]. The sensitivity of ELISA tests against a series of serum antigens ranges from 7 to 94%, and the specificity from 41 to 100%. Faecal culture, milk ELISA antibody and interferon-γ tests exhibit similar problems [9]. Consequently, repeat testing is required to accurately assess the MAP status of herds. MAP detection in calves and youngstock is particularly challenging due to the long incubation period of MAP, leading to low test sensitivities. Indeed, the sensitivity of milk ELISA tests increases almost linearly for cows from 2 to 5 years of age [10]. Furthermore, faecal culture is often able to detect MAP-infected cattle within 6 months of MAP exposure, but detecting infected cattle between this point and the later stages of infection is challenging due to the impacts of cell-mediated and antibody responses on shedding [11]. Given this situation, there is a need for an improved diagnostic test that would allow farmers to identify MAP-infected youngstock and enable more informed management decisions to be made; improved herd management may help in the elimination of MAP infections from affected herds. 

Some studies have suggested the potential of “omic” approaches in relation to MAP diagnostics. Proteomic analysis of cattle serum with advanced MAP infections and control cattle highlighted 32 differentially expressed proteins [12]. Likewise, miRNA analysis using the NanoString technology identified four miRNAs (miR-1976, miR-873-3p, miR-520f-3p and Mir-126-3p) capable of successfully distinguishing between moderately/severely MAP infected cattle and control cattle [13]. Another approach is focused on detecting metabolite changes that are products of complex interactions within the cell and between the cell and its surrounding environment [14]. Such changes can be determined using metabolomic techniques that provide a snapshot of the cells’ responses to stress [15]. The high-throughput capabilities of metabolomics [16], combined with advances in analytical chemistry and metabolite data analysis have increased the use of metabolomics in research [14]. An example of the diagnostic potential of metabolomics is provided by *Mycobacterium tuberculosis* (*M. tuberculosis*) infections of humans. Here, a metabolite signature could predict whether *M. tuberculosis*-exposed individuals will develop symptoms or remain unaffected up to 2 years post exposure with a sensitivity of 69% and a specificity of 75% [17]. 

Metabolomics has yet to be used extensively to examine the effects of MAP infections [18]. Previous sera-based studies of experimentally inoculated Holstein–Friesian calves using ^1^H nuclear magnetic resonance (NMR) spectrometry showed metabolite changes suggestive of energy deficits as well as elevated lipid metabolism and protein turnover [19]. Likewise, Holstein cattle of mixed ages which were categorized as infectious or infected and analysed using direct analysis in real time coupled with high-resolution mass spectrometry (DART–HRMS), showed altered fat metabolism and decreased energy intake, compared to control cattle [20]. However, as with many other omic biomarkers [12,13], none have been commercially developed into a diagnostic method.

This study aimed to identify novel metabolomic biomarkers for MAP by examining the effects of natural MAP infections on the metabolomic profile of Holstein–Friesian heifers, with key changes correlated with changes in the antibody-based detection of selected MAP antigens. Monthly serum samples from naturally MAP-infected and control heifers, aged between 1 month and 19 months were analysed by flow infusion electrospray ionization–high-resolution mass spectrometry (FIE–HRMS), and metabolites whose levels were significantly affected were identified. Crucially, the levels of some of the targeted metabolites were correlated with antibody detection of MAP antigens. We highlight potential metabolite biomarkers for subclinical MAP infections and some likely biological changes that they reflect.

## 2. Results

**Figure 1:**
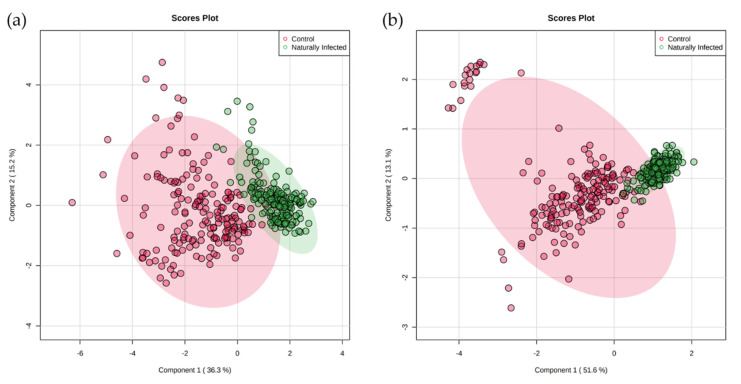
Partial least-squares discriminant analysis (PLS-DA) for naturally MAP-infected and control heifers in the (**a**) negative ionization and (**b**) positive ionization modes. The light red and green ellipses represent 95% confidence intervals.

FIE–HRMS assessments were based on biobanked sera from MAP-infected and age-matched control heifers, aged between 1 month and 19 months. Partial least-squares discriminant analysis (PLS-DA) of the serum metabolite profiles derived from both negative (Figure 1a) and positive ionization modes (Figure 1b) discriminated between naturally MAP-infected and control heifers, with minimal overlap between the 95% confidence intervals for each class. Control heifers (red ellipses) showed greater variation compared to MAP-infected heifers (green ellipses).

**Figure 2:**
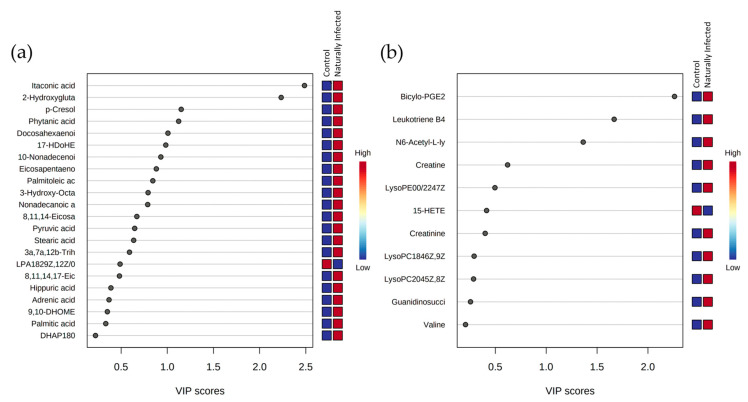
Variable importance in projection (VIP) score plots based on two components from the partial least-squares discriminant analysis (PLS-DA) of metabolites differentially expressed in naturally MAP-infected and control heifers in the (**a**) negative ionization mode *m/z* and (**b**) positive ionization mode *m/z*.

The sources of variation as indicated by PLS-DA were identified using two approaches. In one, variables of importance for the projection (VIP) score plots from two components from PLS-DA were used to indicate metabolites which could discriminate between MAP-infected and control heifers (Figure 2). These indicated that itaconic acid, 2-hydroxyglutaric (Figure 2a), biocylo-PGE2, leukotriene B4 and N6-acetyl-L-lysine (Figure 2b) were particularly prominent sources of variation, in each case showing increases within MAP-infected cattle, compared to controls. 

**Figure 3:**
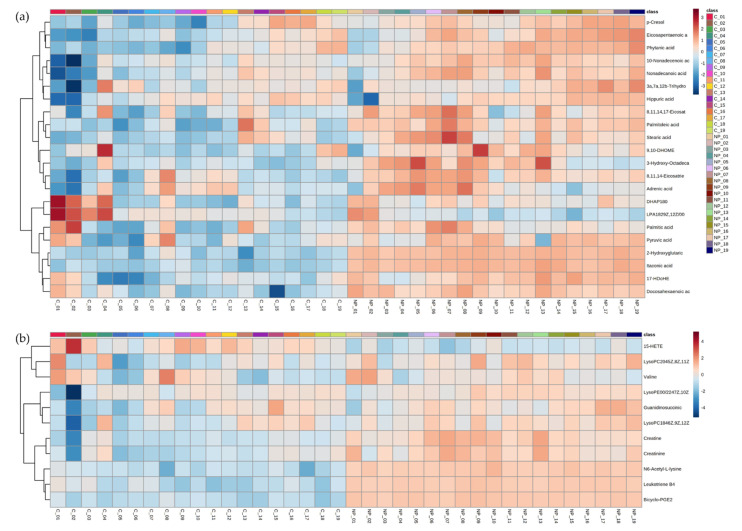
Hierarchical cluster analysis (HCA) of the major metabolite changes differentiating naturally MAP-infected from control heifers between ~1 month and 19 months of age in the (**a**) negative ionization and (**b**) positive ionization mode; C = control heifer, NP = naturally MAP-infected heifer.

Additionally, the experimentally relevant variables were identified by time-series two-way ANOVA (*p* < 0.05), corrected for FDR (false discovery rate). These levels of metabolites were compared using HCA (hierarchical cluster analysis) and showed some variation within naturally MAP-infected and controls groups (Figure 3) in the initial stages of the experiment that could be attributable to different stages of growth and development. However, all heifers, irrespective of their infection status, showed more consistent metabolite accumulation patterns from 14 months of age onwards. The effects of MAP infection were assessed using repeated-measures ANOVA and visualized using estimated marginal means plots. Additionally, analyses indicated 29/33 metabolites and 28/33 metabolites being significantly affected by time and MAP status*time (Supplementary Tables S1 and S2), respectively. Of the 33 identified metabolites, 17 belonged to the fatty acyl class, 14 to the fatty acids and conjugates sub-class, and 3 to the eicosanoids sub-class (Supplementary Table S2). To better understand the biological relevance of the metabolomic changes within MAP-infected heifers, the 33 metabolites were assessed by pathway enrichment analyses based on MSEA (metabolite set enrichment analysis) using ORA (over-representation analysis) (Supplementary Figure S1a,b). This indicated that “alpha linoleic acid and linoleic acid metabolism” and “arachidonic acid metabolism” pathways were significantly enhanced.

**Figure 4:**
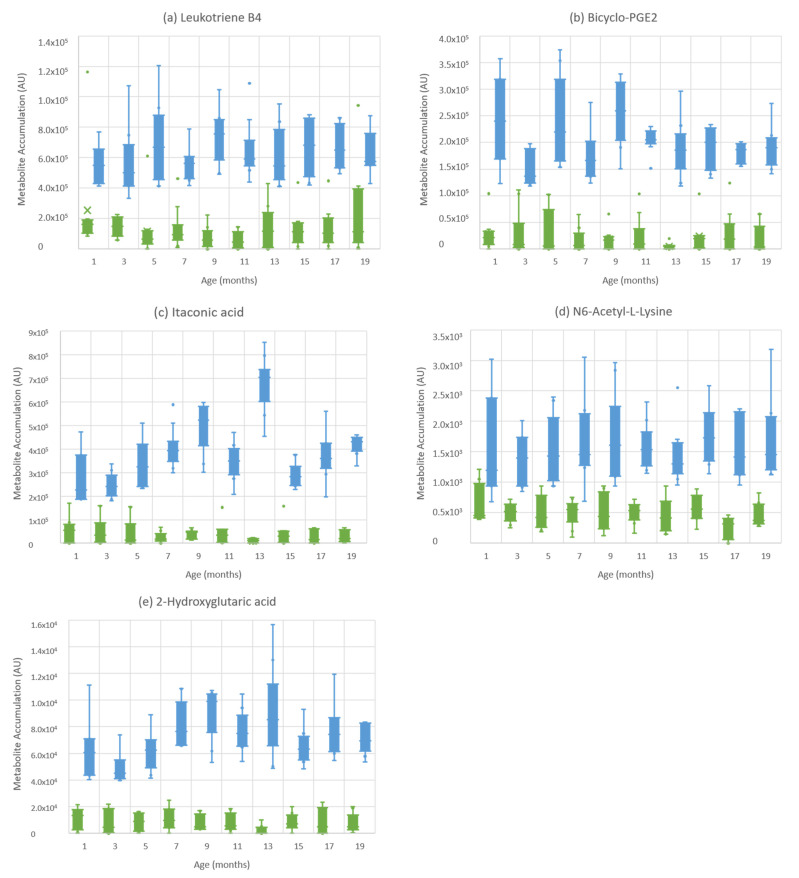
Box and whisker plots of metabolites which displayed minimal overlapping between naturally MAP-infected and control heifers, aged ~1 month to 19 months. (**a**) Leukotriene B4; (**b**) bicyclo-PGE2; (**c**) itaconic acid; (**d**) N6-acetyl-L-lysine (**e**) 2-hydroxyglutaric acid. Blue boxplots = naturally MAP-infected heifers, green boxplots = control heifers.

The five metabolites identified by VIP scores and ANOVA as being particularly prominent sources of variation displayed minimal overlap between MAP-infected and control heifers, as indicated by box and whisker plots (Figure 4). These metabolites were selected for random forest assessments and showed very low classification errors between naturally MAP-infected and control heifers from 14 months of age, of 0.0000 and 0.0189, respectively (Table 1). Based on an optimal cut-off at 0.274577, this model had sensitivity and specificity values of 1.0 and 1.0, respectively. Assessments of the five metabolites for their potential as MAP infection biomarkers were based on area under the curve (AUC) and indicated values >0.889, in the range of 0.889–1.000, at 19 months of age (Supplementary Table S2).

**Figure 5:**
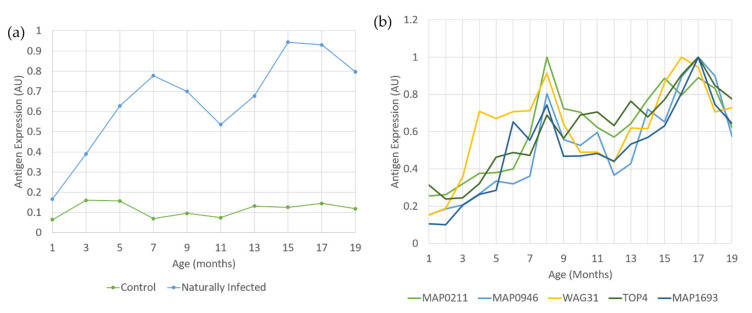
(**a**) Expression of WAG31 in naturally MAP-infected and control heifers between ~1 month and 19 months of age and (**b**) expression of MAP0211, MAP0946, WAG31, TOP4 and MAP1693 in naturally MAP-infected heifers between ~1 month and 19 months of age.

**Figure 6:**
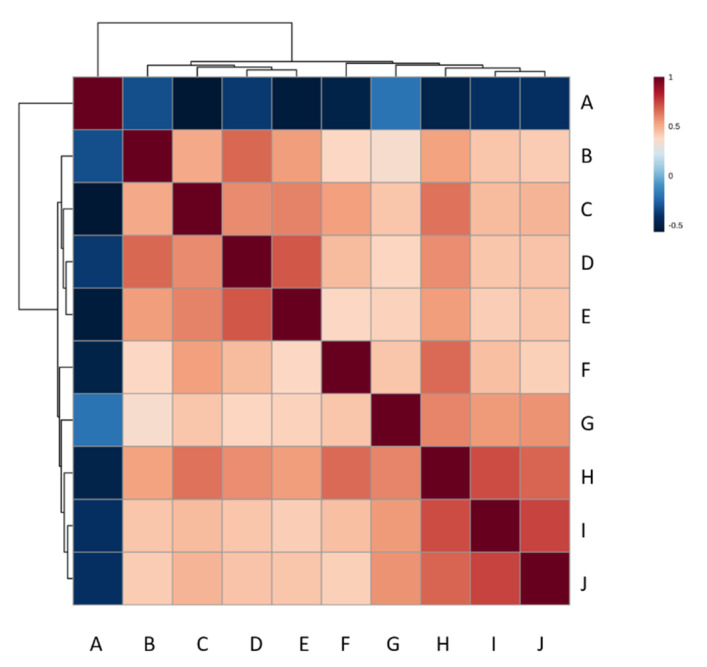
A heatmap of the Pearson’s correlation coefficients produced by comparing metabolites significantly affected by natural MAP infections and MAP-related antigens, whereby −0.4 < correlation co-efficient > 0.4. Positive correlations are shown in red, negative correlations are shown in blue. A = LPA (18:2 (9Z,12Z)/0:0), B = p-cresol, C = Hippuric acid, D = Eicosapentaenoic acid, E = Phytanic acid, F = WAG31, G = TOP4, H = MAP1693, I = MAP0211, J = MAP0946.

Pearson’s correlation analysis was employed to examine the relationship between the 33 metabolites significantly altered by MAP and the antibody responses to 13 antigen/antigen combinations as detected by ELISA (Table 2). Unlike other MAP antigens, data were available for antibody responses to MAP_1889c (Wag31) in both MAP-infected and control heifers. The controls showed some increases in Wag31 antibodies but, as Wag31 is a highly conserved protein, this could reflect responses to environmental mycobacteria. However, serum antibody responses to Wag31 were consistently higher in naturally MAP-infected heifers (Figure 5a). The reduction in Wag31 antibody levels between 10 months and 12 months of age did not correspond to faecal shedding in 6/10 naturally MAP-infected heifers at 13 months of age. MAP0211, MAP0946 and TOP4 displayed similar, but less prominent, patterns to those of Wag31 (Figure 5b). Correlations with the metabolites involved 429 analyses. Of these, 19 proved to be significantly correlated (*p* < 0.05, −0.4 < correlation co-efficient > 0.4), 15 were positively correlated, and 4 were negatively correlated (Figure 6, Supplementary Figure S2). The levels of lysophosphatidic acid (LPA) (18:2 (9Z,12Z)/0:0) was significantly lower in naturally MAP-infected heifers (Supplementary Tables S1 and S2) and displayed a significant, negative correlation with antibody responses to MAP antigens (Figure 6, Supplementary Figure S2). Meanwhile, eicosapentaenoic acid (EPA), p-cresol, hippuric acid and phytanic acid significantly accumulated in MAP-infected heifers (Supplementary Tables S1 and S2) and demonstrated a significant, positive correlation with binding to MAP antigens (Figure 6, Supplementary Figure S2).

## 3. Discussion

Classically, Johne’s disease progression is divided into four stages [2]. The first initial silent stage is typified by no clinical symptoms. During the subclinical stage, it is possible to detect MAP shedding. However, crucially, clinical signs are frequently absent until 2 to 6 years post-infection during the clinical and advanced clinical stages of the disease [5]. There is therefore a need to develop new, sensitive and specific tests for MAP, during this incubation period. IFN-γ tests assess the cell-mediated response associated with early infection but demonstrate poor sensitivity in cattle at 12 to 24 months of age [21]; therefore, their utility during the incubation period is very limited. Faecal culture, antibody ELISA and IFN-γ have been shown to detect MAP in experimentally infected calves, but the results were inconsistent over time [22,23,24]. Furthermore, the peptide-mediated magnetic separation (PMMS)-phage assay has demonstrated the ability to detect viable MAP cells within MAP-exposed mature cattle, but this method requires validation [25]. 

Few studies have used omic techniques to study MAP, particularly metabolomics, despite their ability to demonstrate the dynamics of infection and highlight potential diagnostic biomarkers. Previous studies examining the metabolomics of MAP using NMR and DART–HRMS highlighted significant energy deficits alongside altered fat metabolism in experimentally and MAP-infected cattle [19,20]. In this study, we observed five metabolites which discriminated between naturally MAP-infected and control heifers consistently from 1 to 19 months of age (Figure 4); clearly, these metabolites will require validation with further experimentation. Additionally, FIE–HRMS offered the possibility of obtaining more metabolomic data, providing greater insight into biochemical changes seen in cattle with MAP progression.

### 3.1. Early Changes in Bioenergetic Pathways Suggest Shifts towards Immunomodulatory Pathways

As the clinical phases of Johne’s disease are typified by progressive weight loss, it is perhaps unsurprising that our metabolomic investigations should find changes in bioenergetic metabolism in MAP-infected heifers. However, the key changes identified, including 2-hydroxyglutaric acid and itaconic acid, did not appear to be particularly linked to the derivation of reductants such as NADH, as neither glycolysis- nor TCA cycle-linked metabolism appeared to be affected. The reported changes in TCA metabolites, which could be a reflection of the more potent impact of artificially inoculated MAP infection, were previously noted [19]. In our naturally infected cattle, these changes seemed to be linked to the modulation of the immune system. 

The compound 2-hydroxyglutarate is produced from the TCA intermediate α-ketoglutarate using isocitrate dehydrogenase (IDH) 1 or 3-phosphoglycerate dehydrogenase [26]. Due to its structural similarity to α-ketoglutarate, it can also be produced, at low levels, from malate dehydrogenase (MDH) (which converts malate to oxaloacetate) or lactate dehydrogenase [27]. The accumulation of 2-hydroxyglutarate is a feature of glioblastoma and acute myeloid leukaemia and has been suggested to contribute to carcinogenesis through epigenetic changes and altered immune responses [28]. In cancer, increases in 2-hydroxyglutarate arise through mutations in IDH1 but can also increase physiologically with hypoxia and shifts to acidic pH [29]. CD8+ T cells produce millimolar amounts of 2-hydroxyglutarate, a process mediated by hypoxia inducible factor 1 alpha, when T cell receptors are triggered. The application of 2-hydroxyglutarate has been shown to enhance CD8+ proliferation [30]. Thus, 2-hydroxyglutarate has been suggested to be an “immunometabolite”, mostly likely influencing whether CD8+ cells form memory cells [27]. The rapid elevation of 2-hydroxyglutarate with MAP infection could reflect similar immunometabolic roles which are initiated early in the infection process. 

Itaconic acid is produced from the decarboxylation of cis-aconitic acid within the TCA cycle by the immune-responsive gene 1 protein [31]. Biochemically, itaconic acid can lead to shifts in metabolism through the inhibition of succinate dehydrogenase and, via linkage to co-enzyme A, inactivates the mitochondria coenzyme B12 to influence amino acid and fatty acid metabolism. These metabolic changes may be linked to the roles of itaconic acid in driving proinflammatory effects and macrophage activation [32]. As itaconic acid was significantly elevated in MAP-infected heifers throughout the study (Figure 4), this is evidence of an early reprogramming of the bioenergetic metabolism toward pro-inflammatory responses to infection.

### 3.2. Differential Processing of Eicosanoids Are Features of Subclinically MAP-Infected Cattle

The early initiation of inflammatory events following MAP infection is also suggested by the fact that two of the five metabolites shown to have rapid and persistent increases were eicosanoids (Supplementary Table S2). Pathway analysis comparing gene levels within MAP-infected and control cattle also identified eicosanoid signalling as the top pathway [33]. As examples of eicosanoids, prostaglandins are synthesized from arachidonic acid to prostaglandin (PG) G2 and prostaglandin endoperoxide (PGH) by prostaglandin G/H synthase, (also known as cyclooxygenase, [COX]), before being converted into a range of eicosanoids including PGE2, PGD2 and PGF2a [34]. Bicyclo prostaglandin E2 (bicyclo-PGE2), which we showed to be elevated in MAP-infected heifers, is a stable breakdown product of PGE2, and this feature can be used to estimate PGE2 metabolism which can be difficult to measure. PGE2 increases were also observed in *Mycobacterium bovis* (*M. bovis*)-infected cattle [35] and in humans infected with *M. tuberculosis* [36]. Another eicosanoid, LTB4 was significantly elevated in naturally MAP-infected heifers (Figure 4). LTB4 recruits neutrophils to infected areas and promotes the production of inflammatory cytokines [37]. In leukotriene biosynthesis, arachidonic acid is converted to 5-hydroperoxyeicosatetraenoic acid (HPETE) and then to leukotriene A4 (LTA4) by 5-lipoxygenase (ALOX5) [38]. Within MAP-infected macrophages, ALOX5 is downregulated, but COX2 is elevated between 8 and 24 h post-infection, and ALOX5, along with other immune-related genes, is downregulated 6 months post MAP inoculation [39]. Similarly, within our data, LTB4 levels were also temporarily reduced at approximately 6 months of age (Figure 4). 

Linolenic and α-linoleic acid can feed into the production of EPA, which, in turn, leads to arachidonic acid biosynthesis. It was therefore highly relevant that EPA was significantly elevated within naturally MAP-infected cattle (Supplementary Tables S1 and S2) and that the linolenic acid and linoleic acid metabolism pathway was one of two significantly enriched pathway within MAP-infected heifers (Supplementary Figure S1a). This EPA increase is likely to reflect an increased flux towards the production of eicosanoids. Equally, the discrete roles of EPA should be considered, as this molecule affects a broad range of innate and adaptive immune cells [40]. Notably, EPA exhibits inhibitory effects on IL-10 production within dendritic cells [41], T cells [42] and B cells [43]; IL-10 is a key cytokine in early-phase MAP infections [44]. Moreover, macrophages treated with EPA prior to *M. tuberculosis* infection demonstrated enhanced bacterial growth by inhibiting TNF-α secretion [45]. The positive correlation between EPA and antibody responses to the MAP antigens MAP1693, MAP0211, MAP0946 and Wag31 (Figure 6, Supplementary Figure S2) could indicate that EPA effects aid in MAP proliferation; it would be interesting to test if this is also linked to the inhibition of TNF-α secretion.

### 3.3. Key Metabolites Suggest Uraemic Changes That May Be Indicative of Th1–Th2 Switches

The levels of metabolites within the amino acid, peptide, and analogues subclasses (class, carboxylic acids and derivatives), creatine, creatinine, guanidinosuccinic acid, valine and N6-acetyl-L-lysine were increased in MAP-infected heifers (Supplementary Tables S1 and S2). This was partially in contrast to previous studies. Tata et al. [20] reported an increase in creatine/creatinine in MAP-infected and infectious cattle but a decrease in urea within infectious cattle. However, de Buck et al. [19] demonstrated a significant increase in urea but a decrease in creatinine within MAP-inoculated cattle. This lack of consistency can only be addressed in further studies but could reflect dietary differences. The elevated creatine, creatinine and guanidinosuccinic acid levels may be attributable to increases in arginine. Creatine and creatinine are synthesized indirectly from arginine [46], and guanidinosuccinic acid is produced via the transamidination of arginine [47] in response to elevated urea levels inhibiting urea cycle enzymes [48,49]. 

P-cresol may also be linked to elevated uraemia [50]. P-cresol is a uraemic solute produced from tyrosine and phenylalanine by intestinal bacterial fermentation [51,52]. Whilst p-cresol has not been previously linked to mycobacterial infections, it could have effects that are deleterious to the host. Numerous studies in humans have shown detrimental effects on the immune system, including, the inhibition of phagocyte activity [53] and impaired leukocyte trans-endothelial migration [54]. Interestingly, p-cresyl sulphate, a sulphate-conjugate of p-cresol, downregulates the Th1 (tending to be pro-inflammatory) immune response [55]. In line with this hypothesis, there was a positive correlation between p-cresol and antibody responses with the detection of the MAP antigens MAP1693, MAP0211 and MAP0946 (Figure 6, Supplementary Figure S2) 

Given that we observed metabolomic effects linked to arginine metabolism, it is worth highlighting the immunological roles of this important amino acid. Arginine is required for the proliferation of T lymphocytes [56] and influences the levels of a functional T cell receptor [57]. Further, it affects the cytotoxicity of monocytes and NK cells in vitro [58]. Clearly, a major role for arginine is in acting as the substrate for NO synthesis by inducible NO synthase (iNOS) in macrophages and neutrophils. [59]. iNOS levels are induced in leucocytes in response to IFNγ, directly linking arginine, NO and the Th1 response. The diversion of arginine to creatine and creatinine guanidinosuccinic acid in MAP infections could influence NO production and, possibly, be suggesting an increased dominance of the T helper 2 (Th2)-mediated humoral response [26] as the infection progresses. This would align with the Th2 response being associated with MAP faecal shedding [60], the presence of multibacillary lesions [61] and the development of clinical symptoms [62]. 

De Buck et al. [19] observed increases in lysine, and we also observed higher levels of N6-acetyl-L-lysine in MAP-infected heifers throughout the study (Figure 4). N6-acetyl-L-lysine is a post-translationally modified version of lysine. Lysine shares the same transport systems with arginine and if lysine levels are elevated, it can reduce arginine uptake and thereby affect NO synthesis by iNOS [63]. Therefore, the elevation in lysine could also be an indicator of suppressed NO production in macrophages [64]. However, to assess if our observed changes in amino acid metabolism were indicative of a classical Th1–Th2 shift, will require correlations with actual measurements of cytokine changes.

## 4. Materials and Methods

### 4.1. Animal Samples

The animal experiments were approved by the Animal Welfare Body of WBVR (MAP infected animals, permit number 299-47053-07/99-01), and the Animal Welfare Body of Utrecht University (control heifers, permit number 0202.0806) in accordance with the Dutch regulations on animal experimentation. 

Sera from 10 naturally MAP-infected and 10 age-matched control heifers were sampled for a period of 40 and 22 months, respectively. Control Holstein–Friesian heifer calves were sourced at an age of approximately 14 days from MAP-negative (control heifer calves) herds. The herd status of MAP-negative farms was indicated from the culture of faecal samples obtained from animals that were at least 2 years old and were sampled once or twice yearly for a period of at least 8 years as part of a Dutch national MAP surveillance program. In addition, these heifers were MAP-negative at necropsy. 

Naturally MAP-infected heifers were not experimentally exposed to MAP but demonstrated MAP-positive faecal culture results during routine testing. Heifers displayed MAP-positive faecal culture results at the following ages (number of positive heifers between brackets); 7 months (one), 13 months (six), 16 months (one), 39 months (one) and 48 months (one). Faecal samples from naturally MAP-infected heifers were incubated on Lowenstein–Jensen (LJ) growth medium, and the presence of MAP was confirmed using IS900 PCR at Wageningen Bioveterinary Research in Lelystad, the Netherlands. Faecal samples from control heifers were processed using the routine diagnostic MAP faecal culture system at the Veterinary Health Service laboratory (Royal GD), Deventer, the Netherlands. All infected heifers were housed in conventional facilities of Wageningen Bioveterinary Research in Lelystad, the Netherlands; all control heifers were housed in conventional facilities at the faculty of Veterinary Medicine, Utrecht University, Utrecht, the Netherlands.

### 4.2. Antibody Tests

The detection of serum antibodies to diagnostic MAP antigens by ELISA has been published previously [65,66]. The MAP antigens used in this study are listed in Table 2. The ELISA tests were performed as described previously [66], except that a different dose of antigen was used in coating the plates. Thus, each single recombinant antigen was coated at a final concentration of 10 μg/mL whilst the recombinant antigens mix (TOP4) was coated at a concentration of 40 μg/mL. 

**Table 2:** Details of recombinant MAP antigens, antigen mixes and commercially available ELISA tests.

Antigen.		Antigen Description (Uniprot for Recombinants)
MAP_0210c	Ag1del1	PirG; P36 or erp (Exported Repeated Protein)
MAP_0211	-	Glf; UDP-galactopyranose mutase activity
MAP_0946	-	Uncharacterized protein; DNA binding
MAP_1272	-	NLPC_P60 domain-containing protein; integral membrane protein
MAP_1693c	-	Peptidyl-prolyl cis–trans isomerase
MAP_1889c	Antigen 84	Wag31; Cell wall synthesis protein
MAP_2609	Ag 7	Uncharacterized protein; heme binding
MAP_2821	-	Endoribonuclease L-PSP
MAP_2942c	Ag2, mpt53	Mpt53; oxidoreductase activity
TOP4	TOP4	Mix of MAP_0210c, MAP_1272, MAP_1693c and MAP_2609
-	ID-Vet	Commercially available absorbed ELISA (ID-Vet): ID Screen^®^ Paratuberculosis Indirect
-	Pourquier	Commercially available absorbed ELISA (Pourquier): IDEXX^®^ Pourquier Paratuberculosis ELISA Antibody

### 4.3. Untargeted Metabolite Fingerprinting by Flow Infusion Electrospray Ionization-High-Resolution Mass Spectrometry (FIE–HRMS)

Sera were prepared as described by Beckmann et al. [67], with minor amendments. Samples were defrosted on ice, vortexed for 5 s, and 200 µL was pipetted into 1520 µL of a pre-chilled solvent mix (methanol/chloroform [4/1]) containing 1 micro-spoon of glass beads (<160 μM glass beads (Sigma-Aldrich Ltd., Gillingham, UK)). Samples were then vortexed for 5 s, shaken for 15 min at +4 °C and kept at −20 °C for 20 min. Following centrifugation at 21,000× *g* and 4 °C for 5 min, 100 µL of the plasma supernatant was transferred into mass spectrometry vials along with 100 µL of methanol/water [70/30]. Samples were stored at −80 °C until analysis using flow infusion electrospray ionization–high-resolution mass spectrometry (FIE–HRMS). For each sample, 20 µL was injected into a flow of 60 µL per minute of water/methanol, at a ratio of 70% water and 30% methanol, using a Surveyor flow system into a Q Exactive plus mass analyser instrument with a UHPLC system (Thermo Fisher Scientific©, Bremen, Germany). Data acquisition for each serum sample was done alternating the positive and negative ionization modes, throughout four different scan ranges (15–110 *m/z*, 100–220 *m/z*, 210–510 *m/z*, 500–1200 *m/z*) with an acquisition time of 2 min. The derived data matrices are provided in Supplementary Tables S3 and S4.

### 4.4. Statistical Analysis

Metabolomic data were analysed using MetaboAnalyst 4 [68]. Data were subjected to interquartile range-based filtering, log10 transformations and Pareto scaling. Time series analyses used FDR-adjusted two-way ANOVA tests to identify *m/z*, which significantly (*p*-values < 0.05) differed between experimental classes. VIP scores (>2) following multivariate analyses were also used to indicate *m/z* which discriminated between the classes. PLS-DA was used to visualise the differences between the experimental classes. Random forest was used as an alternative multivariate classification test. This uses a confusion matrix to estimate how often a given *m/z* variable would provide an estimate of the classification error. The major sources of variation were displayed using unsupervised HCA. AUC based on sensitivity and specificity estimates was used to determine the accuracy of the target *m/z* as potential biomarkers. 

Significant *m/z* were identified based on accurate mass (5 ppm) using the DIMEdb database based on their ionised masses, molecular formula and the Bovine Metabolome Database [69]. All isotopes/adducts were considered. MSEA using ORA was used to highlight key biochemical pathways. Correlation analysis between metabolites and antibody binding to MAP antigens was performed using Pearson’s correlation coefficient.

## 5. Conclusions

Metabolomic analysis of naturally MAP-infected heifers and control heifers demonstrated the ability of untargeted FIE–MS to examine the metabolic processes of mycobacterial infections. Our findings showed a clear differentiation of metabolites between naturally MAP-infected and control heifers aged between 1 month and 19 months, including five metabolites which were significantly elevated within naturally MAP-infected heifers throughout the trial. These metabolites can identify MAP-infected heifers irrespective of their age, thus, highlighting their potential as biomarkers for subclinical MAP infections. The identified metabolites also highlight the potential effects of MAP infection on primary metabolism and the immune system. As these findings are preliminary, future work could include validating these metabolites using independent samples, as well as assessing the levels of these metabolites within clinically MAP-infected cattle demonstrating symptoms and verifying the specificity of these metabolites by comparison with *M. bovis*-infected cattle. 

## 6. Patents

We have submitted a patent entitled “Biomarkers for *Mycobacterium avium paratuberculosis*”, which is under review (Intellectual Property Office Patent Application No. 2018541.9). The Patent Filing Institution is Aberystwyth University. 

## Figures and Tables

**Table 1:** Cross validation results of the random forest assessments for leukotriene B4, bicyclo-PGE2, itaconic acid, 2-hydroxyglutaric acid and N6-acetyl-L-lysine in heifers aged between 14 months and 19 months.

	Control	Naturally Infected	*Class Error*
Control	52	1	0.0189
Naturally Infected	0	60	0.0000

## Data Availability

The metabolomics and metadata reported in this paper are available in the supplementary data.

## Supplementary Materials

The following are available online at https://zenodo.org/record/5364099#.YS_Zso5KiUk, Figure S1: Significantly enriched pathways in naturally MAP-infected heifers in the (a) negative ionization mode and (b) positive ionization mode, Figure S2: A heatmap of the Pearson’s correlation coefficients produced by comparing metabolites significantly affected by natural MAP infection and MAP-related antigens. Positive correlations are shown in red, negative correlations are shown in blue, Table S1: Metabolite expression within naturally MAP-infected and control heifers, Table S2: Metabolites differentially expressed in naturally MAP-infected and control heifers at 19 months of age, Table S3: Raw data, negative ionization mode, Table S4: Raw data, positive ionization mode.
